# The effects of preoperative chemotherapy on isolated tumour cells in the blood and bone marrow of gastric cancer patients

**DOI:** 10.1038/sj.bjc.6603904

**Published:** 2007-08-14

**Authors:** P Kolodziejczyk, A Pituch-Noworolska, G Drabik, J Kulig, A Szczepanik, M Sierzega, A Gurda, T Popiela, M Zembala

**Affiliations:** 1 1st Department of Surgery, Jagiellonian University Medical College, 40 Kopernika Street, Krakow 31-501, Poland; 2 Department of Clinical Immunology, Jagiellonian University Medical College, Krakow 31-501, Poland

**Keywords:** gastric cancer, isolated tumour cells, neoadjuvant chemotherapy

## Abstract

Recent studies in breast cancer suggest that monitoring the isolated tumour cells (ITC) may be used as a surrogate marker to evaluate the efficacy of systemic chemotherapy. In the present study, we have investigated the effects of preoperative chemotherapy on ITC in the blood and bone marrow of patients with potentially resectable gastric cancer. After sorting out the CD45-positive cells, the presence of ITC defined as cytokeratin-positive cells was examined before and after preoperative chemotherapy. The patients received two courses of preoperative chemotherapy with cisplatin (100 mg m^−2^, day 1) and 5-fluorouracil (1000 mg m^−2^, days 1–5), administered every 28 days. Fourteen of 32 (44%) patients initially diagnosed with ITC in blood and/or bone marrow were found to be negative (responders) after preoperative chemotherapy (*P*<0.01). The incidence of ITC in bone marrow was also significantly (*P*<0.01) reduced from 97 (31 of 32) to 53% (17 of 32). The difference between patients positive for ITC in the blood before (*n*=7, 22%) and after (*n*=5, 16%) chemotherapy was statistically insignificant. The overall 3-year survival rates were 32 and 49% in the responders and non-responders, respectively (*P*=0.683). These data indicate that preoperative chemotherapy can reduce the incidence of ITC in patients with gastric cancer.

Isolated tumour cells (ITC) in blood and bone marrow of cancer patients are observed even in early stage disease (Maehara *et al*, 1998). Although their prognostic and predictive applications have been thoroughly examined in patients with breast cancer, the exact nature of ITC in gastric cancer remains to be determined. Depending on the methods used, ITC are detected in up to 42 and 80% of blood and bone marrow samples, respectively (Bonavina *et al*, 2001; Chen *et al*, 2004). Moreover, surgical manoeuvres increase detection rates of ITC in many malignancies, including gastric cancer and thus preoperative evaluation seems to reflect more accurately the actual extent of the disease (Miyazono *et al*, 2001; Ikeguchi and Kaibara, 2005).

There is a growing body of evidence that chemotherapy may affect the outcomes of patients with resectable gastric cancer (Carrato *et al*, 2005). However, unlike tumour response criteria in unresectable cancer, no reliable surrogate markers permitting the immediate assessment of the efficacy of chemotherapy have been developed so far. The idea of using ITC as such a marker is gaining an increasing attention since verification of other potential compartments for cancer cells, for example, lymph nodes, is usually impossible before surgery. The hypothesis that monitoring the presence of ITC after chemotherapy may be clinically justifiable has been recently verified in breast cancer and has produced promising results (Slade *et al*, 2005; Drageset *et al*, 2006; Fehm *et al*, 2006). No such observations are available for resectable gastric cancer so far.

The purpose of this study was to investigate the effects of preoperative chemotherapy on the presence of ITC in blood and bone marrow of patients with potentially resectable gastric cancer.

## MATERIALS AND METHODS

### Patients

Two hundred and sixty-eight patients with gastric cancer admitted between 2001 and 2005 were examined for the presence of ITC in blood and bone marrow (Popiela *et al*, 2005). Thirty-two consecutive patients positive for ITC in either blood or bone marrow and diagnosed with a potentially resectable tumour were selected for this study. There were 21 (66%) males and 11 (34%) females with a median age of 62 years (95% confidence interval (CI) 56–64). In all cases, the preoperative diagnosis of adenocarcinoma of the stomach was confirmed by gastroscopic biopsy. The Bioethics Committee of the Jagiellonian University approved the protocol of this study and all patients gave informed consent before taking part in the study.

### Chemotherapy

All patients received two courses of preoperative chemotherapy with cisplatin and 5-fluorouracil (5-FU), administered every 28 days. Each cycle consisted of continuous intravenous infusion of 5-FU for 5 days at a dose of 1000 mg m^−2^ and intravenous cisplatin on day 1 at a dose of 100 mg m^−2^. Within 21 days from the completion of preoperative chemotherapy, all patients were scheduled for gastrectomy. At laparotomy, the primary lesion was found unresectable in five cases (peritoneal spread in four patients and infiltration of coeliac trunk in one patient), while the remaining 27 patients underwent gastric resection. Nineteen of these patients received another two cycles of postoperative chemotherapy. The remaining eight were not given chemotherapy due to non-advanced lesions (stage IA *n*=4, stage IB *n*=2) or poor performance status following surgery (*n*=2).

### Detection of ITC

Peripheral blood samples (20 ml) and bone marrow aspirates (5 ml) from the iliac crest were taken before chemotherapy and immediately before surgery. Control blood samples were collected from clinically healthy blood donors (*n*=8) and bone marrow from patients with suspected idiopathic thrombocytopaenia (*n*=8). Pelleted cells from blood and bone marrow samples were incubated with an excessive amount of lysing solution (Becton Dickinson Biosciences, San Jose, CA, USA) for 10 min, repeated 3–4 times to remove erythrocytes. The remaining cells were washed in phosphate-buffered saline (PBS) and adjusted to the concentration of 1 × 10^7^ cells ml^−1^ in PBS. Subsequently, the cells were stained with monoclonal mouse anti-human CD45 (phycoerythrin labelled) antibodies (DAKO, Glostrup, Denmark) and sorted into CD45^+^ and CD45^−^ populations using flow cytometry (FACS Vantage SE, BD Biosciences, Bedford, MA, USA) equipped with the TurboSort (BD Biosciences) option and Aerosol Protection System (Flexoduct International ApS, Greve, Denmark). The Innova Enterprise II ion laser (Coherent, Santa Clara, CA, USA) operating at 488 nm was used as a light source. Sorting was performed using a 70 mm nozzle tip with a drop drive frequency of 65 kHz, 1.5-drop envelopes and a ‘normal’ sorting mode. Sorted CD45^−^ cells were collected into polystyrene Falcon 2057 tubes (BD Biosciences) precoated with fetal calf serum and maintained in a refrigerated bath recirculator (Neslab Instruments, Portsmouth, NH, USA). About 1 × 10^6^ of CD45^−^ cells (1 × 10^6^ cells ml^−1^) were used to prepare slides. The slides were dried, fixed with a mixture of ethanol and acetone (1 : 1 v v^−1^), and then stained for 30 min with A45-B/B3 monoclonal antibodies (5 *μ*g ml^−1^) (Micromet GmbH, Germany), which recognise common epitopes of cytokeratins (CK) including CK 8, 18 and 19. Subsequently, the slides were washed and stained for 30 min with goat anti-mouse IgG-FITC-labelled antibodies (DAKO). After washing with PBS, the slides were assayed within 2 days. The CK^+^ cells were identified by two independent investigators under a BX60 fluorescent microscope (Olympus, Tokyo, Japan) and documented with a DP10 camera (Olympus). At least 300 cells were examined per slide. The samples were regarded as positive when at least three CK^+^ cells were found per slide. Accordingly, patients were classified into CK^+^ and CK^−^ groups. After two cycles of chemotherapy, patients negative for CK^+^ cells, both in blood and bone marrow, were regarded as responders. Non-responders were classified as the presence of CK^+^ cells in either blood or bone marrow.

### Statistical analysis

The *χ*^2^ test or the Mann–Whitney *U*-test was used to detect differences between the groups with regard to categorical or ordinal parameters, respectively. Survival data were analysed according to the Kaplan–Meier method and the log-rank test was used to detect differences between individual groups. The differences at *P*<0.05 were regarded as statistically significant. Statistical analysis was performed using the SPSS v.14 (SPSS Inc., Chicago, IL, USA) software package.

## RESULTS

### Bone marrow status preoperatively and during follow-up

A detailed description of ITC before and after preoperative chemotherapy is shown in Table 1. Isolated tumour cells were eradicated in four of seven patients initially presenting with CK^+^ cells in blood. However, three patients negative for tumour cells in blood before chemotherapy were subsequently found to be CK^+^. Fifteen of 31 patients with ITC in bone marrow became CK^−^ and only one patient changed his status from CK^−^ to CK^+^. The overall response rate was 44% since 14 of 32 patients initially diagnosed with ITC in the blood and/or bone marrow were found to be CK^−^ after preoperative chemotherapy. There were no chemotherapy-related deaths and no patient developed grade 3 or 4 toxicity as defined by the WHO criteria.

**Figure 1:**
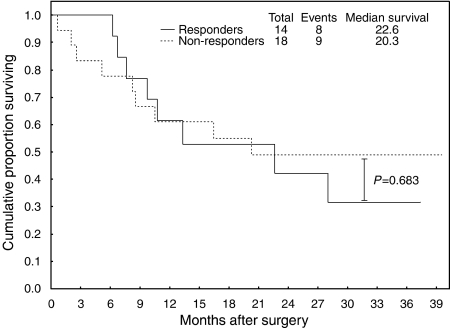
Overall survival by response to neoadjuvant chemotherapy.

Except a higher incidence of diffuse-type cancers in the responders group (11 of 14 *vs* 7 of 18, *P*=0.024), there were no significant differences between responders and non-responders with respect to the clinicopathological variables (Table 2). The median follow-up period was 15.3 months (95% CI 9.7–28.4) and 17 patients died within 36 months following surgery (Figure 1). The overall 3-year survival rates were 32 and 49% in the responders' and non-responders' groups and the corresponding median survival time was 22.6 and 20.3 months, respectively (*P*=0.683).

## DISCUSSION

The results of the present study support an accumulating body of evidence indicating that ITC in blood and/or bone marrow can be found in patients with either early or advanced gastric cancer. However, unlike other reports, we have specifically demonstrated that preoperative chemotherapy with cisplatin and 5-FU significantly reduces the incidence of CK^+^ cells in bone marrow.

Immunocytochemical staining with monoclonal antibodies is commonly accepted as an adequate method to examine the presence of ITC in blood and bone marrow. Such assays using CK markers for epithelial tumours can detect a single tumour cell against the background of millions of normal cells. Nevertheless, no definite diagnostic standard has been established so far and at least several ‘enrichment’ methods have been developed to increase the chance of detecting malignant cells. Moreover, some authors examining ITC in the bone marrow of patients with upper gastrointestinal tumours subject to thoracotomy advocated that rib segment resections rather than iliac crest aspirates should be used to achieve reliable results (O'Sullivan *et al*, 1999; Bonavina *et al*, 2001; Mattioli *et al*, 2001). As no sample obtained from healthy individuals has produced a positive result for CK^+^ cells, we believe that the specificity of our assay is sufficient. Moreover, the use of immunohistochemical and morphological criteria, as opposed to PCR-based techniques reduces the risk of false-positive results (Jung *et al*, 1999). On the other hand, application of A45-B/B3 antibodies that recognise common epitopes of CKs increases the possibility of detecting tumour cells. Furthermore, the applied methodology, based on sorting out all leukocytes (CD45^+^) and searching for CK^+^ cells among the remaining CD45^−^ population, may ‘concentrate’ the presumptive tumour cells and increase the rate of detection (Pituch-Noworolska *et al*, 1998, 2007).

The prognostic implications of ITC in patients with tumours of the gastrointestinal tract are controversial. In gastric cancer, ITC in the bone marrow were either significantly associated with overall survival (Jauch *et al*, 1996), disease-free survival (Gretschel *et al*, 2006), or were devoid of any prognostic value (de Manzoni *et al*, 2002). However, the presence of ITC is interesting not only from the prognostic point of view, but also for their potential predictive value. The idea of ITC being used as a surrogate marker for monitoring the effects of chemotherapy is gaining increasing attention in breast cancer (Slade *et al*, 2005; Drageset *et al*, 2006; Fehm *et al*, 2006). However, the information available for gastrointestinal malignancies is scarce. Surprisingly, some authors suggested that palliative chemotherapy might even increase the incidence of ITC. Of 42 stage IV patients with colorectal cancer, 62.5% patients with distant metastases and 14.3% with locally advanced diseases were positive for ITC in blood using a RT-PCR assay for CK 20 (Staritz *et al*, 2004). After the first cycle of chemotherapy, the detection rates in the latter group increased to 62.5%. Moreover, patients responding to chemotherapy showed an increase in detection rates from 28.5 before to 71.4% after chemotherapy.

To our knowledge, no study evaluating the effects of preoperative chemotherapy on ITC in blood and bone marrow of gastric cancer patients has been undertaken. In a study carried out by Bonavina *et al* (2001), the proportion of ITC in the bone marrow of patients with oesophageal cancer was similar irrespective of whether they received previous neoadjuvant chemotherapy (80.0%, 12 of 15) or not (79%, 26 of 33). Similar findings were reported by O'Sullivan *et al* (1999) for 50 patients undergoing potentially curative resections of oesophageal or oesophagogastric malignancies. The prevalence of ITC in rib marrow of patients given neoadjuvant chemotherapy (87%, 13 of 15) was similar to that in patients who underwent surgery only (89%, 31 of 35). Contrary results were reported in a study on oesophagogastric cancer (Ryan *et al*, 2004). Isolated tumour cells were detected in the bone marrow in 42% of patients (23 of 55) who received neoadjuvant therapy plus surgery, and in 67% of patients (34 of 51) treated with surgery alone (*P*=0.01). Owing to contradictory results, no clear conclusions can be formulated for gastric cancer. Moreover, the presence of ITC in all these studies was examined only once after the completion of systemic therapy, and thus no exact comparison with the present study could be made. In this study, repeated examinations of blood and bone marrow before and after neoadjuvant chemotherapy provided evidence that chemotherapy reduced the presence of ITC in 44% of the patients initially positive for blood and/or bone marrow. Nevertheless, CK^+^ cells were still found in more than one-half of the patients. This is not surprising considering the fact that most ITC in the bone marrow are assumed to be in a state of dormancy at the time of chemotherapy, while systemic treatment eliminates only proliferating cells (Slade *et al*, 2005). Contrary to clinical trials in breast cancer, we were unable to demonstrate any survival benefit of preoperative chemotherapy in the responders group. This finding is unexpected and challenges the idea of ITC as a surrogate marker for monitoring the efficacy of chemotherapy in gastrointestinal malignancies. However, our pilot study involved a relatively small number of patients and was not aimed at evaluating survival benefits. Possibly, more recent regimens of chemotherapy developed for gastric cancer or novel therapies also directed against quiescent cells could further increase eradication rates for ITC.

In conclusion, preoperative chemotherapy with cisplatin and 5-FU can significantly reduce the incidence of ITC in patients with resectable gastric cancer. However, it was not associated with any clear survival benefit. Therefore, other clinical trials specifically evaluating this issue are required.

## Figures and Tables

**Table 1:** Incidence of ITC before and after chemotherapy

	**Blood**		**Bone marrow**		**Blood and/or bone marrow**	
**ITC**	**Before**	**After**	**Before**	**After**	**Before**	**After**
Negative	25 (78%)	27 (84%)	1 (3%)	15 (47%)	0 (0%)	14 (44%)
Positive	7 (22%)	5 (16%)	31 (97%)*	17 (53%)*	32 (100%)*	18 (56%)*

* *P*<0.01, Wilcoxon-matched pairs test.

**Table 2:** The clinicopathological variables in patients responding and non-responding to neoadjuvant chemotherapy

	**Responders (*n*=14)**	**Non-responders (*n*=18)**	***P***
*Gender*			
Male	7 (50%)	14 (78%)	0.100^a^
Female	7 (50%)	4 (22%)	
Age, mean (95% CI)	64 (57–70)	60 (54–65)	0.323^b^
			
*Tumour location*			
Upper third	1 (7%)	5 (28%)	
Upper/middle third	4 (29%)	2 (11%)	0.176^a^
Middle third	2 (14%)	3 (17%)	0.176^a^
Middle/lower third	3 (21%)	5 (28%)	0.176^a^
Lower third	1 (7%)	3 (17%)	0.176^a^
Whole stomach	3 (21%)	0 (0%)	0.176^a^
			
*Lauren's type*			
Intestinal	3 (21%)	11 (61%)	0.024^a^
Diffuse	11 (79%)	7 (39%)	
			
*Depth of infiltration* c			
T1	1 (7%)	3 (17%)	0.756^a^
T2	2 (14%)	4 (22%)	
T3	6 (43%)	6 (33%)	
T4	5 (36%)	5 (28%)	
			
*Nodal status*			
Negative	4 (29%)	10 (56%)	0.126^a^
Metastatic	10 (71%)	8 (44%)	
			
*Stage* c			
I	2 (14%)	5 (28%)	0.768^a^
II	1 (7%)	2 (11%)	
III	5 (36%)	5 (28%)	
IV	6 (43%)	6 (33%)	
			
*Gastrectomy*			
No	2 (14%)	3 (17%)	0.854^a^
Yes	12 (86%)	15 (83%)	
			
*Postoperative chemotherapy* d			
No	3 (25%)	5 (33%)	0.962^a^
Yes	9 (75%)	10 (67%)	

a *χ*^2^ test.

b Mann–Whitney *U*-test.

c AJCC classification of 2002.

d Patients who underwent gastrectomy.
